# Retraction: Carbohydrate response element binding protein (ChREBP) modulates the inflammatory response of mesangial cells in response to glucose

**DOI:** 10.1042/BSR20180767_RET

**Published:** 2026-05-06

**Authors:** Yan-Jun Wang, Ying Zhao, Jin-Cheng Wang, Yan Chen

**Affiliations:** Department of Endocrinology, The Second Hospital of Jilin University, Changchun 130000, China

**Keywords:** carbohydrate response element binding protein, cytokine, diabetic nephropathy, inflammation

This article is being retracted from *Bioscience Reports* at the request of the Editor-in-Chief and the Editorial Board. This follows the receipt of a notification from the authors, alerting the Editorial Board to an error in Figure 3B and requesting to correct the aforementioned Figure.

Similarities were also noted by a reader between both the Figure 3B B-actin and ChREBP bands of this paper and the Figure 2 Ad-GFP/GADPH and Ad-FBXO22/KLF4 (after a horizontal flip) bands respectively from the publication by Tian et al. (2015) DOI: 10.18632/oncotarget.4082.

The authors provided a replacement for Figure 3B; however, as no original raw data could be provided for any of the western blots of the paper, concerns have not been alleviated, and the Editorial Board stands by the decision to retract.

